# Measuring impact of New Mexico prekindergarten on standardized test scores and high school graduation using propensity score matching

**DOI:** 10.1186/s40723-023-00112-9

**Published:** 2023-03-19

**Authors:** Jon R. Courtney, Janelle Taylor Garcia, Jacob Rowberry, Nathan Eckberg, Sarah M. Dinces, Clayton S. Lobaugh, Ryan T. Tolman

**Affiliations:** State of New Mexico, Legislative Finance Committee, Program Evaluation Unit, 325 Don Gaspar Ave, Suite 101, Santa Fe, NM 87501 USA

**Keywords:** Prekindergarten, Early childhood education, Principal component analysis, Education, Preschool

## Abstract

The long-term impact of prekindergarten programs is an important consideration given the trend of dedicating more resources to these programs. However, long-term impact of prekindergarten programs is not well-understood and recent studies have shown preschool effectiveness can vary across states and programs. A state run prekindergarten program in New Mexico was examined using propensity score matching to minimize selection bias. The research revealed a number of long-term impacts corresponding with prekindergarten participation for the cohort including a 9.7 percentage point increase in high school graduation rates and improved reading and math proficiency at third, sixth, and eighth grades. Considerations for future research and challenges in implementing prekindergarten programs are discussed.

New Mexico is one of many states faced with challenges in child development and student outcomes. New Mexico ranks the lowest in the U.S. in terms of educational outcomes for children (Oakes et al., 2020). New Mexico also shows above average rates of families living in poverty as well as immigrants (U.S. Census Bureau, 2020b). Students facing socioeconomic challenges, including experiencing poverty and learning English as a second language, are considered “at-risk” (Winsler et al., 2014). Student performance is significantly disparate among at-risk students, when compared to their economically stable or English-speaking peers (New Mexico Legislative Finance Committee, 2017). On average, New Mexico schools provide students with at least a year’s worth of academic learning as measured by growth on state standardized assessments over time; however, many at-risk students start academically behind their peers and never catch up; a phenomenon referred to as the achievement gap (New Mexico Legislative Finance Committee, 2017). In New Mexico, low-income students consistently have lower average scores on state standardized assessments than their non-low-income peers (New Mexico Legislative Finance Committee, 2017). Previous research has found that, high poverty schools in New Mexico see one in four children that cannot recognize a single letter upon entry into kindergarten (New Mexico Legislative Finance Committee, 2014). In 2005, the New Mexico legislature passed the Pre-Kindergarten Act. The Pre-Kindergarten Act established a state-run preschool program for 4-year-old children. This Act had many goals including a focus on school readiness.

For decades, researchers have found emerging evidence that preschool programs have the potential to provide long-term educational and societal benefits. Beneficiaries include participating children, and taxpayers (Decker-Woodrow et al., 2020). Early evidence supporting the effectiveness of prekindergarten programs has come from longitudinal studies of cohorts participating in the Perry Preschool Project, the Abecedarian Project, and in Chicago Child–Parent Centers (Berrueta-Clement et al., 1984; Campbell et al., 2002; Reynolds et al., 2018).

The Perry Preschool Project (PPP) is an early intervention program for disadvantaged children. Research examining the impact of PPP found students who participated in the program had higher educational achievement compared to those not enrolled in the program. Program participants also showed higher earnings and a higher rate of employment in adulthood (into the participant’s 40s). Participants of the PPP were more likely to own homes, be married, and have less involvement with the criminal justice system (Belfield et al., 2006). Based on these data, the societal return on investment for preschool expenditures was estimated at $12.90 for every $1 invested (Belfield et al., 2006). In addition, Nores et al. (2005) found that 12% more PPP participants had completed high school by age 40 than comparison group nonparticipants.

The Abecedarian Project is a full-day and full-year program for at-risk children from birth through age five in North Carolina. Researchers found similar long-term positive effects between PPP and the Abecedarian Project. Children who were enrolled in the Abecedarian Project subsequently experienced higher lifetime earnings, better health outcomes, lower incidences of smoking, and lower public assistance enrollment than the children who were not enrolled in the program (Barnett & Masse, 2007; Campbell et al., 2014). Regarding impact on high school completion, although Campbell et al. (2002) found slightly higher graduation rates for preschool participants in the Abecedarian Project than nonparticipants (70% versus 67%), the difference was not statistically significant. Similarly, Campbell et al. (2012) found similar high school and GED completion rates for Abecedarian Project treatment and control groups (89% versus 82%), but a higher proportion of the treatment group obtained a high school diploma rather than a GED (83% versus 72%).

The Chicago Child–Parent Center Program (CCPCP) is a center-based early intervention that provides educational and family-support services to disadvantaged children. Research on CCPCP found positive social outcomes for program participants including higher educational attainment, higher lifetime earnings, and lower criminal justice costs (Reynolds et al., 2002, 2018). Reynolds et al. (2007) found that Chicago Child–Parent Center Program participants had a 7.7% higher rate of high school completion than similar nonparticipants in a comparison group.

In response to evidence supporting the positive outcomes from preschool participation, states across the country continue to create and expand state funding for preschool programs. According to the National Institute for Early Education Research (NIEER), 34% of 4 years in the United States received preschool services from their state in 2019 (National Institute for Early Education Research [NIEER], 2020). Over the past 17 years, states across the country increased preschool funding by $6.1 billion (254%). States have also increased preschool enrollment for 4 years by 798 thousand (137%). In 2002, states’ spending on preschool was $2.4 billion. In 2019, states’ spending was $8.5 billion to fund 1.38 million 4 years (NIEER, 2003; NIEER, 2020). Given the significantly increasing investments in preschool programs, it is important for researchers to examine the effects of specific state-funded preschool programs as these programs differ from their predecessors in many ways. State-funded preschool programs rely on evidence-based research and practices used in other successful programs. However, each program is unique and delivers different outcomes. Variance of outcomes exists, because each program will have different influences affecting the progression of those outcomes. Previous literature has established state-funded preschool effectiveness can vary across states and across programs based on several factors. Factors include curriculum, teacher training, classroom size, and program structure (Phillips et al., 2017). The generalizability of outcomes found in PPP, the Abecedarian Project, and other early preschool projects is a matter for discussion. These programs have never been replicated fully among current publicly funded programs. Notably, the estimated cost to replicate PPP or the Abecedarian Project exceeds the amount of capital that any state currently allocates per child. The per child annual cost of PPP is estimated to be about $20,000 in current dollars, and between $16,000 to $40,000 for Abecedarian, far exceeding the U.S. average per child annual cost of $5374 (Heckman et al., 2010; NIEER, 2020).

Regarding long-term impacts of early childhood education programs, McCoy et al. (2017) provide a meta-analysis of early childhood education studies finding participation in early childhood education leads to significant reductions in special education placement, grade retention, and increases in high school graduation. In addition, Schweinhart et al. (2012) found that 58% of participants in Michigan’s Great Start Readiness Program, a state-funded preschool program, graduated from high school on time compared with 43% of demographically similar nonparticipants. Schweinhart et al. (2012) also found these statistically significant impacts were greater for students of color. Despite this evidence, Cannon et al. (2017) identifies a need for more studies that conduct long-term follow-up to examine sustainability of early program impacts, identifying the fact that the bulk of study growth of early childhood program evaluation has focused on following study participants for short increments. Cannon et al. (2017) further state that these types of long-term studies could facilitate the use of benefit cost analysis. Of course, not all research studies find longitudinal impacts of early childhood education.

The results through sixth grade of a longitudinal randomized control study of the effects of a scaled-up, state-supported Tennessee prekindergarten program (TN-VPK) showed a negative effect in third grade, and a larger negative effect in sixth grade. Data through sixth grade showed that the children randomly assigned to attend TN-VPK had lower state achievement test scores in third through sixth grades than control children, with the strongest negative effects in sixth grade. A negative effect was also found for disciplinary infractions, attendance, and receipt of special education services, with null effects on retention (Durkin, et al., 2022).

Some state funded prekindergarten programs have sufficient operations to measure impact, resist fade-out effects, and prove to be cost-sustainable (or near sustainable). Preschool programs that meet the aforementioned criteria include New Jersey’s Abbott Preschool Program (NJAPP), Boston Prekindergarten (BPK), Maryland’s Extended Elementary Education Program (EEEP), North Carolina’s More at Four (NCMF) program (Minervino & Pianta, 2013), as well as the state of New Mexico’s prekindergarten (NMPK) program. Research assessing the impacts of NJAPP, BPK, EEEP and NCMF has found promising effects in academic performance (Barnett et al., 2013; Forry et al., 2013; Peisner-Feinberg & Schaaf, 2010; Weiland & Yoshikawa, 2013). More recent propensity score matching analysis continued to find positive associations between participation in the Abbott Preschool Program and student achievement. Specifically, the more recent research again found that the Abbott Preschool Program was significantly associated with positive effects on statewide academic assessments, reduced grade retention, and reduced special education need lasting through 10th grade (Barnett & Jung, 2021). Achievement effects were also larger for students who participated in the program for 2 years rather than 1 year.

New Jersey funds three preschool programs. Among these programs, exists the largest and most intensive, NJAPP. NJAPP targets children in the highest poverty school districts and is open to 3-year-old and 4-year-old students. Research has shown that children entering kindergarten who spent 2 years in NJAPP saw 50% larger gains in vocabulary, 2% larger gains in print awareness, and 14% larger gains in math, when compared to students who spent 1 year in the program (Barnett et al., 2013). Subsequent analysis showed persistent gains with NJAPP participants through the fifth grade (Barnett et al., 2013). According to Barnett et al., (2013), long-term effects were equivalent to a plus 10-percentile boost in state test scores. Barnett’s research also shows lower special education placement rates, and lower grade retention, corresponding to NJAPP participation.

Boston’s prekindergarten (BPK) program targets all children in Boston. Two-thirds of BPK participants live in poverty. Most recently, research findings show that participation in BPK corresponds with increased college attendance, higher rates of students taking SAT, and increased graduation rates (Gray-Lobe et al., 2021). However, impact is not consistently evident on standardized tests. Previous studies examining the impact of prekindergarten on children in early grade levels found increases in children’s vocabulary, early reading, and numeracy, when assessed at the end of the school year.^1^ Participants who live in poverty, as well as, those whose primary language is Spanish showed larger than average gains. Findings from an assessment on BPK participation showed significantly more advanced skills in math, literacy, and language skills among BPK participants, in comparison with nonparticipants. This is true through the third grade. Third grade Massachusetts Comprehensive Assessment System (MCAS) scores, showed that 43% of BPK participants scored proficient or advanced in English and Language Arts, compared to 34% of nonparticipants. Studies on third grade MCAS English Language Arts scores, as well as, studies on BPK indicate program persistence through third grade (Weiland & Yoshikawa, 2013).

Students who participated in Maryland’s Extended Elementary Education Program (EEEP) full-day program showed higher preparedness for kindergarten, as demonstrated with higher academic performance and a greater number of students meeting kindergarten-reading benchmarks (Forry et al., 2013). Maryland has made rapid and large gains in academic achievement through fourth grade. Participation in North Carolina’s More at Four, (NCMF) program corresponds with significant academic achievement differences, and moderate to large effect sizes, between program participants and nonparticipants; with effects also persisting into third grade (Peisner-Feinberg & Schaaf, 2010). Participation in NCMF was associated with higher sustained math and reading test scores for children in poverty, but not for other participants.

In 2017, a study of Tulsa Oklahoma’s Universal Prekindergarten program (TOUP), sought to answer the question of whether attending a strong school-based prekindergarten produces lasting beneficial effects for program participants through middle school (Gormley et al., 2018). This TOUP research focused on students who began kindergarten in the 2006–2007 school year. Findings showed strong positive effects on academic success that diminish over time, but do not completely disappear. There was also a modest statistically significant positive relationship between prekindergarten enrollment and standardized math scores. Reading scores did not show relationships of true significance. In addition, this TOUP study found a significant relationship between prekindergarten participation and increased enrollment in honors courses 8 years later. Grade retention results were substantial. Students attending prekindergarten were 7% less likely to repeat a grade; this was true for all subgroups in the study. Two well-known, decades-long studies followed the progression of children who attended experimental preschool programs in the 1960s and 1970s. The High/Scope Perry Preschool Program studied prekindergarten programs in Michigan, and the Abecedarian Project studied programs in North Carolina (Galinsky, 2006). Research from Tulsa found that students in this prekindergarten study did not have significantly higher grades or fewer disciplinary suspensions.

In 2005, the New Mexico Legislature established the statewide voluntary New Mexico prekindergarten program (NMPK), which provides services to 3-year-old and 4-year-old children through public schools, tribes, pueblos, Head Start centers, and licensed private providers.^2^^,^ 3 From 2005 to 2020, the Public Education Department (PED) and the Children, Youth and Families Department (CYFD) jointly oversaw and administered the state’s NMPK services. PED oversaw all NMPK services provided through public schools, while CYFD regulated all other NMPK service providers, most of which were privately owned and operated. In 2019, the New Mexico Legislature created the Early Childhood Education and Care Department (ECECD) to oversee all early childhood programs and administer state NMPK programs in coordination with PED (New Mexico Legislative Finance Committee, 2019a, 2019b).^4^ From state fiscal year^5^ 2005–2006 (FY06) to FY20, New Mexico increased NMPK funding by $84.5 million (2,113%) and NMPK enrollment by 10,919 children (709%). For FY20, the New Mexico Legislature appropriated $88.5 million to serve 12,459 3-year-old and 4-year-old children (New Mexico Legislative Finance Committee, 2019a, 2019b, 2020).

Researchers from National Institute for Early Education Research (NIEER) evaluated the first 4 years of the NMPK program. The NMPK provided half-day, center-based services to 4 years (Hustedt et al., 2007). In NIEER’s evaluation of first year program implementation, NMPK met five out of 10 benchmarks, such as class sizes and staffing ratios (NIEER, 2006), which improved in subsequent years to meeting eight out of 10 benchmarks (NIEER, 2008). Further evaluations found when compared to nonparticipants, children who participated in NMPK had higher reading achievement, math achievement, and early literacy outcomes by kindergarten, providing an estimated $6 return on every $1 invested in the program (Hustedt et al., 2007, 2008, 2009a, 2009b, 2010). Specifically, Hustedt et al. (2007) found that NMPK participants had 54% growth in vocabulary scores, 40% more growth in early math scores, and 26% more growth in early print comprehension by kindergarten than comparison group children. In a 2021 study, *Impacts of the New Mexico PreK Initiative by Children’s Race/Ethnicity*, data were pooled from five successive cohorts of children (total *N* = 5218) using regression-discontinuity models to estimate the impacts of participating in NMPK on young children’s language, literacy, and math skills at kindergarten entry. The study found positive, statistically significant impacts of NMPK for each of these academic domains. Due to the high level of diversity in the sample, it was also possible to examine NMPK impacts separately for White, Hispanic, and Native American children. The largest impacts were found for White and Hispanic children, with less consistent and more modest impacts for Native American children. These findings suggest that while NMPK generated academic benefits for children, not all groups of children benefited equally, and further information is needed to understand the reasons for these differences (Hustedt et al., 2021). Program evaluators from the New Mexico Legislative Finance Committee (NMLFC) have conducted studies on NMPK. Findings show that participation in state prekindergarten programs is associated with higher reading and math proficiency levels through primary and secondary school (New Mexico Legislative Finance Committee, 2016, 2017, 2018; 2019b).

In this study, we assess how participation in NMPK corresponded with later performance on state standardized assessments and high school graduation. This assessment is an effort to build upon previous research about NMPK and contribute to the overarching research literature on the longitudinal effectiveness of state-funded preschool programs (New Mexico Legislative Finance Committee, 2020).

## Research questions

Our team focuses on two research questions: (1) does participation in NMPK improve academic outcomes on state standardized assessments in English language arts and math? (2) Does participation in NMPK increase the likelihood of high school graduation? We developed two hypotheses. The first hypothesis was that participants in the inaugural 2006 cohort of NMPK would have better outcomes on state standardized assessments in later grade levels. Assessment outcomes are measured by comparing the percent of students scoring proficiently on English language arts and math assessments, to statistically similar nonparticipants. Our second hypothesis predicts that participants in the inaugural 2006 cohort of NMPK would have a higher graduation rate from high school than statistically similar nonparticipants.

## Method

This retrospective study utilized a causal comparative design and archival student data to observe the effects of attending prekindergarten in New Mexico between the treatment and comparison groups on standardized test scores and graduation rates. New Mexico’s longitudinal public education data system, housed at PED, provided data on the inaugural prekindergarten class of school years 2005–2006 (SY06). Data obtained includes student standardized test scores assessing performance of mathematics and English language arts along with data regarding high school graduation. Data from the New Mexico Standards-Based Assessment (SBA) were obtained for the 2009–2010 (third grade), 2012–2013 (sixth grade), and 2014–2015 (eighth grade) academic years. Finally, Partnership for the Assessment of Readiness for College and Career (PARCC) assessment data was obtained for the 2017–2018 academic year (11th grade),^6^ and graduation data were obtained for the 2018–2019 academic year.

A randomized control trial was not an option for this study. Therefore, to minimize selection bias, propensity score matching (PSM) was utilized to determine a control group for analysis. The propensity score matching method used five variables: ethnicity (A = Asian; B = Black; C = Caucasian; H = Hispanic; I = Native American or Alaskan Native), FRL (1 = eligible for free or reduced lunch; 0 not eligible for free or reduced lunch), English as a second language (1 = receiving support services for English language acquisition; 0 = not receiving support services), special education (1 = receiving special education support services; 0 = not receiving special education support services), and the school each individual attended. Although additional covariates for propensity score matching are preferred (e.g., parental income), these were the only demographic variables collected by the state’s education department, and have been demonstrated to impact student performance (New Mexico Legislative Finance Committee, 2017). The authors used PSM over propensity score weighting as the latter method is used to ensure samples are representative of specific populations (Benedetto et al., 2018). The sample used in the current study represented all participants with the program initially being targeted to Title I school districts. With the potential of NMPK becoming universal in New Mexico, future studies may want to consider the use of propensity score weighting. This study also explored alternative methods, such as multilevel modeling (New Mexico Legislative Finance Committee, 2020). Like propensity score matching (PSM), multilevel modeling can control for factors likely influencing outcomes (e.g., FRL status, EL status, SPED status, school attended), isolating the treatment effect of attending prekindergarten. Since the multilevel modeling analysis found similar results, the results of the causal comparative design from PSM is reported here for simplicity.

### Participants and cohort development

The treatment population consisted of all 1509 students in the inaugural New Mexico prekindergarten class of school years 2005–2006. To arrive at the study’s treatment sample, one duplicate record was identified in the public education data system and removed from the analysis. Of the 1509 unique prekindergarten participants who were registered for prekindergarten, 84 students were withdrawn by a family member and 14 students were inactivated by New Mexico prekindergarten, leaving 1411 students who were enrolled in prekindergarten for the entire academic year. Due to high rates of attrition and mobility, treatment participants were excluded from analysis of student outcomes at each cohort (third, sixth, eighth, and 11th grade) if they had not completed either a reading or math test. Fewer cohort test score records were available as time progressed. Cohort numbers for comparisons of each grade range from 878 (third grade) to 525 (11th grade). There are several potential attributions to lack of test scores including missed school. For analysis of student graduation rates, 588 treatment group participants were excluded, since they were no longer counted in New Mexico’s public education graduation cohort for school years 2018–2019, leaving 823 prekindergarten students in the final graduation cohort for the propensity score model analysis. For testing the effect of prekindergarten on student outcomes, the potential comparison comprised of New Mexico public education students who completed both a standardized reading and math test in third grade (school years 2009–2010), sixth grade (school years 2012–2013), eighth grade (school years 2014–2015), or 11th grade (school years 2017–2018). For testing the effect of prekindergarten on student graduation rates, the potential comparison group consisted of 25,480 students from the 2018–2019 graduation cohort who had not attended NMPK. Demographic information of the pre-matched treatment and comparison groups utilized for analysis of graduation rates is reported in Table 1.

**Table 1:** Pre/post-match balance results for analysis of graduation rates

Pre-match	Comparison (*N* = 25,480)		Treatment (*N* = 823)		Standardized difference
Covariates	Mean	SD	Mean	SD	
FRL	63.38%	0.48	75.82%	0.43	0.273
ELL	31.03%	0.46	41.19%	0.49	0.213
SPED	14.49%	0.35	9.60%	0.29	0.151
Ethnicity					
Asian	1.90%	0.14	0.12%	0.03	0.178
Black	2.36%	0.15	0.85%	0.09	0.120
Caucasian	24.68%	0.43	12.03%	0.33	0.331
Hispanic	60.64%	0.49	65.01%	0.48	0.900
Native American	10.42%	0.31	21.99%	0.41	0.318
Number of High Schools Represented	125		90		
Average SMD					0.311

Post-match	Comparison (*N* = 823)		Treatment (*N* = 823)		Standardized difference
Covariates	Mean	SD	Mean	SD	
FRL	76.18%	0.43	75.82%	0.43	0.009
ELL	41.19%	0.49	41.19%	0.49	< 0.001
SPED	9.60%	0.29	9.60%	0.29	< 0.001
Ethnicity					
Asian	0.12%	0.03	0.12%	0.03	< 0.001
Black	0.61%	0.08	0.85%	0.09	0.029
Caucasian	12.15%	0.33	12.03%	0.33	0.004
Hispanic	64.88%	0.48	65.01%	0.48	0.003
Native American	22.24%	0.42	21.99%	0.41	0.006
Number of High Schools Represented	90		90		
Average SMD					0.007

FRL: free or reduced lunch; ELL: English language learner; SPED: special education; SMD: standardized mean difference; SD: standard deviation

Previous research on student mobility and emigration suggests that more than half of students move schools at least once (Temple & Reynolds, 1999). Mobility and emigration data also show a significant proportion of students moving out of state. These findings reflect similarly in records over this research’s study period. However, there is an absence of a data system that contains information on students leaving the state and students attending private schools. There is also an absence of data about students in other types of schools; including those run by the Bureau of Indian Education (Gasper et al., 2012; Joint Center for Housing Studies, 2020). Due to these absences, patterns of attrition come with a degree of uncertainty. Research has also found that income contributes to attrition through mobility and emigration; lower-income families being more likely to move (Frost, 2020). New Mexico currently ranks 48th in the nation for children living in poverty (U.S Census Bureau, 2020a, 2020b). Students who switch schools are more likely to have negative educational outcomes than students who do not switch schools (Gasper et al., 2012). Many public school students in New Mexico switch schools in early grades. For example, 46% of students who started kindergarten in school years 2012–2013 changed schools by third grade (New Mexico Legislative Finance Committee, 2017). Due to the high degree of mobility and attrition in both the treatment and control groups, PSM was conducted at each testing point (third, sixth, eighth, 11th grade testing, and 12th grade graduation status).

### Propensity scores

Propensity scores commonly are employed as a statistical method to account for confounding effects when random assignment is not possible (e.g., Austin, 2011). Propensity score models attempt to mimic random assignment by calculating the probability of receiving treatment for each individual in both the treatment and control group. Based on a set of observed characteristics (i.e., covariates)—the resulting probability (ranging from 0 to 1) for each individual is the propensity score. Once propensity scores are calculated, there are many techniques utilized as a means of using the scores to control for research biases.

Propensity score matching involves finding a control individual with a propensity score that is equal or nearly equal to the propensity score for each treatment individual (Cleophas et al., 2012). By doing so, a subgroup from the entire control population is matched with the treatment group based on the set covariates that comprise the propensity score. This process approximates the random assignment of individuals into treatment and control groups in a randomized control trial.

To assess the quality of matching, approximation of randomization and covariate balance checks are performed (Beal & Kupzyk, 2014). This consists of validating group differences in observed demographic and baseline characteristics by comparing group means. In a well-balanced propensity score match design, group means for covariates should not be statistically different from each other. Comparison of the control and treatment groups on outcome measures follows propensity score matching. Verification of group balance is assessed by means of a covariance analysis as well as a Chi squared test.

### Matching and PSM score assignment

PSM is used to assign each participant a single score to represent their likelihood of receiving treatment. The propensity score represents all covariates as a single summed score calculated for each student. Matching was performed in the R statistical analysis software using the MatchIT package (Ho et al., 2011). A nearest neighbor one-to-one fixed ratio was performed, where each treatment case was matched to one comparison case. Covariates were matched on all available demographic variables which included ethnicity, FRL, ELL, and special education, as well as high school attended.

As the initial prekindergarten sites targeted at risk communities, demographic characteristics among participants were different than statewide averages.^7^ The treatment and comparison groups did not differ in a statistically significant manner across the covariates, establishing a comparable group was successfully achieved. The mean FRL value for the treatment group was 75.8% compared to 76.2% in the comparison group. As students who receive free and reduced lunch generally have worse academic outcomes (New Mexico Legislative Finance Committee, 2017), this difference between the treatment and comparison groups represents a conservative comparison group for the participation group. The mean ELL value for both the treatment group and comparison group was 41.2%. Hispanic students accounted for a majority of observations, with 65% in the treatment group and 64.9% in the comparison group. Native American students were the next largest ethnic group represented in the treatment and comparison groups (around 22%), followed by Caucasian students (around 12%). Black and Asian students accounted for 1% or less in both the treatment and comparison groups. In both groups, the same set of 90 high schools were represented in the data.

To ensure treatment and comparison groups were appropriately constructed, and subsequent statistical comparison appropriate, covariate balance diagnostics were calculated. Standardized mean difference (SMD) is the most common statistic to assess the balance of covariate distribution between the two groups (Zhang et al., 2019). The *cobalt* package in R was used to compute SMD between the treatment and comparison groups. Covariate imbalance across groups is indicated by SMD values greater than 0.1 (Stuart et al., 2013). The SMD results showed that there were no significant differences across covariates, demonstrating balance between the two groups for each comparison of interest (standardized test scores at third, sixth, eighth, and 11th grade, and graduation outcomes). Results of the propensity score matching for comparison of graduation rates is reported in Table 1. The average SMD was reduced from the pre-match level of 0.311 to the post-match average of 0.007, which indicates a sufficiently balanced match to conduct causal comparative analyses.

## Results

To answer the first research question (Is participating in New Mexico prekindergarten associated with higher rates of reading and math proficiency?), chi-square analyses were conducted for each cohort match at third grade, sixth grade, eighth grade, and 11th grade to compare proficiency rates on reading and math. Because multiple independent significance tests were conducted, a Bonferroni correction was applied to the α-level to control for overall Type I error rate (α = 0.05/8 = *p* < 0.00625). The dependent variable was whether the student scored proficient or not on standardized assessments of reading and math. Table 2 reports descriptives for reading and math proficiency rates for the prekindergarten treatment and matched comparison group, chi-square tests, and associated odds ratios. Prekindergarten participants significantly outperformed matched nonparticipants in third, sixth, and eighth grade on both reading and math assessments, but not in 11th grade. Although prekindergarten participants displayed higher rates of reading proficiency compared to their match comparison group in 11th grade, this difference was nonsignificant after applying the Bonferroni correction. Reasons for not finding an effect for the 11th grade comparison include potential fade out effects as previously discussed, a lack of power due to lower *n* size, or a higher natural variance in math scores as students often have multiple options for math coursework in high school.

**Table 2:** Descriptives and chi-squares comparing prekindergarten treatment participants to matched nonparticipants on reading and math tests

Grade (*n*)	Assessment	Prekindergarten treatment group	Matched comparison group	*χ*^2^ (1)	p^*^	Odds ratio (95% CI lower and upper bound)
		Mean (SD)	Mean (SD)			
3rd (*n* = 878)	Reading	56.5% (0.50)	45.9% (0.50)	19.29	< 0.001*	1.53 (1.26, 1.86)
3rd (*n* = 878)	Math	58.3% (0.49)	47.4% (0.50)	20.62	< 0.001*	1.55 (1.28, 1.88)
6th (*n* = 804)	Reading	43.5% (0.50)	30.7% (0.46)	27.72	< 0.001*	1.74 (1.41, 2.14)
6th (*n* = 804)	Math	39.9% (0.49)	27.6% (0.45)	26.70	< 0.001*	1.74 (1.41, 2.16)
8th (*n* = 723)	Reading	23.4% (0.42)	14.0% (0.35)	20.44	< 0.001*	1.88 (1.42, 2.49)
8th (*n* = 723)	Math	16.2% (0.37)	11.1% (0.31)	7.62	0.006*	1.55 (1.13, 2.13)
11th (*n* = 525)	Reading	36.2% (0.48)	28.8% (0.45)	6.27	0.012	1.40 (1.07, 1.84)
11th (*n* = 525)	Math	4.95% (0.22)	5.9% (0.24)	0.30	0.586	0.83 (0.47, 1.47)

*Denotes significant chi-square test after Bonferroni correction (α = 0.05/8 = 0.00625) was applied due to multiple significance tests

To answer the second research question (Does participating in New Mexico prekindergarten lead to higher graduation rates?), a chi-square test was performed to determine the probability of graduating high school based on participating in prekindergarten. The dependent variable was whether the student in the prekindergarten treatment or matched comparison group graduated high school within 4 years or not. Out of the 823 students in the prekindergarten treatment group, 669 graduated from high school, while 154 did not graduate (mean graduation rate = 81%, SD = 0.39). Among the 823 matched comparison students, 589 graduated high school and 234 did not graduate (mean graduation rate = 72%, SD = 0.45). The chi-square test revealed that there was a significant association between participating in prekindergarten and the probability of graduation high school *χ*^2^(1) = 21.05, *p* < 0.001. Based on the odds ratio, the odds of students graduating high school was 1.73 (1.36, 2.19) times higher if they had attended prekindergarten. Note that researchers also conducted a separate analysis to examine NMPK participation on high school graduation using hierarchical linear modeling yielding similar results (New Mexico Legislative Finance Committee, 2020).

## Discussion

States across the country have created and expanded state funded preschool programs in response to early research on the long-term effects of experimental preschool programs from the 1960s and 1970s. Recent research has explored the impacts of these state funded programs. This paper contributes to finding made in previous literature by exploring how participation in the inaugural 2006 cohort of NMPK influenced standardized test score performance and high school graduation rates. This research is the first study to examine long-term academic impacts of NMPK beginning with third grade performance assessments through high school graduation. Through this research, we also build upon previous research identifying positive effects from NMPK on student academic achievement (Hustedt et al., 2007, 2008, 2009a, 2009b, 2010, 2021).

Our team tested two hypotheses. The first hypothesis predicts that participants in the inaugural 2006 cohort of NMPK would have better outcomes on state standardized assessments through primary and secondary school. Percent of students scoring proficiently on reading and math assessments was used as a metric to assess the outcomes of state assessments. The results revealed that a higher percentage of NMPK participants scored proficiently on standardized reading assessments in third grade, sixth grade, and eighth grade. In addition, a higher percentage of NMPK participants scored proficiently on standardized math assessments than nonparticipants in nearly all observed grades, with the exception of 11th grade. A potential reason for not finding an effect for 11th grade math is that students often have multiple options for math coursework in high school, indicating that students are operating at different academic levels, and a smaller *N* size for that grade. The results support our first hypothesis correct by providing evidence that participation in NMPK is associated with better outcomes on state standardized assessments in both primary and secondary school. Our findings positively contribute to existing literature which indicates that participation in state funded preschool programs can have lasting positive effects on standardized test scores (Barnett et al., 2013; Barnett & Jung, 2021; Pisner-Feinberg & Schaaf, 2010; Weiland & Yosikawa, 2013). However, other current studies have found participation in state funded preschool programs to have insignificant, sometimes negative, effects on standardized test scores throughout the course of a student’s academic progression (Durkin et al., 2022; Gray-Lobe et al., 2021). As previously mentioned, preschool effects can vary across states and programs based on multiple factors ranging from curricula to teacher training (Phillips et al., 2017). This is especially true for long-term effects.

Regarding the impact of high school graduation, New Mexico’s increase in high school graduation of 9.7% is slightly lower than that estimated in McCoy et al. (2017) meta-analysis of 11.4 percentage points, which relied heavily on effects sizes from the previously cited Abecedarian Project, the Chicago Child–Parent Center Program, and the Perry Preschool. Along these lines, for the current study the authors conducted further analysis regarding effect size uses Hedges *g* effect size, a variant of the more common Cohen’s *d*, using the mean difference divided by pooled standard deviation. The calculated effect size of 0.16 can be categorized as a medium effect size (Kraft, 2020), but it is smaller than the larger effect size for high school graduation rates found in the McCoy et al. (2017) meta-analysis (*d* = 0.24 *SD*, 11.4 percentage points). McCoy et al. (2017) point to a potential impacting factor between current programs and those included in their meta-analysis including older programs having unique characteristics including those programs being implemented when alternative care options were limited, the targeting of high-risk children, the inclusion of “wrap-around” care with home-visiting components, and the provision of services for multiple years. These characteristics of older more intensive programs could explain the reason for the slightly lower effect size found for NMPK.

Our second hypothesis predicts that participants in the inaugural 2006 cohort of NMPK would have a higher high school graduation rate when compared to statistically similar nonparticipants. The results of a Chi-squared analysis revealed that NMPK participants had a significantly higher graduation rate (81.3%) than the comparison group of nonparticipants (71.6%). These results support the second hypothesis by showing that preschool participation can help increase a participant’s likelihood of graduating high school. These results are consistent with recent findings, by indicating that participation in state funded preschool is associated with increased high school graduation.

### Limitations and future research

Our team encountered limitations in the number of variables available for PSM. Analysis is limited by the variables that are collected by the state of New Mexico. Notably, available data show an inability to distinguish between children who participate in federally funded head start, privately paid programs, or state funded services. However, the likelihood of children participating in multiple early education programs (e.g., Head Start and NMPK) is quite low as the programs run during the same time of day. In fact, a report from the New Mexico Legislative Finance Committee (2019b) cites oversaturation and competition from multiple program offerings as a potential concern citing the expansion of NMPK as a potential reason that the number of state Head Start slots have decreased by 30% since 2012. In addition, the potential impact from children dually enrolled in NMPK and child care is likely minimal as evidence from publicly funded child care shows 8.6% of children enrolled in NMPK were also enrolled in child care in 2019 (New Mexico Legislative Finance Committee, 2019b). Due to the fact that so few children are dually enrolled in Head Start and NMPK or NMPK and child care, it is unlikely that these programs are responsible for impacts attributed to NMPK. Furthermore, it should be noted that the New Mexico Legislative Finance Committee has found child care participation to have minimal impacts on educational outcomes (New Mexico Legislative Finance Committee (2019a). It is possible that some comparison group children participated in child care or Head Start instead of NMPK, and if these programs had beneficial impacts it might result in a lower effect size for NMPK. Our team also encountered data limitations due to the student attrition rate of New Mexico public schools. This is particularly concerning for future research of prekindergarten cohorts and participants, since public school enrollment has declined substantially because of the COVID-19 pandemic. There is an inability to track students moving into private schools or moving out of the state, causing inability in tracking students’ outcomes for those moving through school systems outside of New Mexico’s public schools. Caution should be taken in generalizing results from the NMPK to other states as New Mexico has a unique population. Prekindergarten programs in other states might be different in terms of curriculum, program structure, and classroom quality as they have different populations to serve and different outcome expectations.

The level of quality of the NMPK program is also a limitation in the current study. NIEER (2006) publishes a quality standards checklist each year including benchmarks around early learning standards, teacher degree and training, class sizes, staff–child ratios and other metrics. In 2006, New Mexico scored a 4.8 out of 10 on this metric signaling the need for additional quality guardrails which have since been added. More recently, NIEER (2021) gave New Mexico a score of 9 out of 10 on this metric. With improved quality, it is reasonable to expect increased impact in subsequent years of program implementation. More specifically, associating these quality elements was not possible in this study but could be in future studies of NMPK. The study of the effects of quality, program design and other program inputs in future studies is essential for understanding why some programs like NMPK see significant long-term gains, whereas other programs, such as Tennessee’s Prekindergarten program do not (Durkin et al., 2022). The difference in outcomes between these two programs also signals that researchers and policymakers should use caution in generalizing results from studies on early childhood education programs (Barnett, 2022). Evaluation of state prekindergarten programs has potential to derive data on classroom quality, which provides additional context for generalization of results. Data on classroom quality were not available for the 2006 inaugural NMPK cohort. However, data on quality, as measured by the Early Childhood Environment Rating Scale-Revised (ECERS-R), is available beginning school year 2008. Research on future NMPK cohorts could leverage quality data and provide further information on the impact of classroom quality on outcomes.

The state of New Mexico has applied for waivers of standardized testing in 2020 and 2021. As a result, these data will be available in 2 years, and will help with understanding how participation in programs affects ELA, math, and science assessment outcomes. Students in select New Mexico districts were asked to complete a kindergarten entry assessment in 2021. Unfortunately, data resulting from this assessment were deemed unreliable due to several reasons, including selection bias. Lower-income students are less likely to return to the public education system during the pandemic. In addition, assessments were taken at home, yielding the potential for students to receive help from parents or caregivers.

Future research should also consider the potential for participation in multiple programs. In 2006, it was rare for a student to participate in multiple programs; however, as program availability has increased, more students are participating in multiple programs. It is of growing importance to examine which programs students are participating in to provide further information for policymakers on decisions regarding expansion of these programs.

Finally, program quality is an area of concern for NMPK. There is variation in the delivery of prekindergarten services. This is exacerbated with two separate agencies providing different systems of governance for NMPK. As a result, measuring program quality varies in method between these two systems. These two agencies have different methods of measuring quality as well as distinct teacher qualifications. Legislative Finance Committee, (2019) includes a complete discussion of differences in quality and should be considered for review by future researchers. Recent incorporation of the two systems under a new umbrella department, referred to as, the Early Childhood Education and Care Department, may help provide more standardization among approaches and metrics of quality. In addition, New Mexico lacks a statutory definition of school readiness. Numerous researchers have taken attempts to define school readiness based on statutory definitions in other states and previous research. Despite these efforts, there remains a need for New Mexico to formally define the expectations of school readiness. Again, the new department has committed to working towards defining school readiness in 2021.

## Conclusion

Our research, exploring NMPK impacts, analyzed student administrative data from the state public education department to compare the long-term academic outcomes of prekindergarten participants with the outcomes of statistically similar nonparticipants who were matched using the propensity score matching technique. The findings indicate that a higher percentage of participants in NMPK later scored proficiently on state standardized assessments in English language arts and mathematics than nonparticipants. Significant differences in state standardized assessment performance were observed in all measured grade levels, except the 11th grade. The present study also found that participants in NMPK also later had a higher graduation rate from high school than nonparticipants.

These findings contribute to the research literature by examining the long-term impact of a state funded prekindergarten program. It is important to assess the long-term effectiveness of state funded preschool programs as states across the country create and expand such programs. The findings are particularly relevant to New Mexico, where policymakers have significantly expanded state funded prekindergarten since the program’s inception in 2006. State funded prekindergarten programs have the potential to improve educational outcomes and reduce achievement gaps. However, factors associated with the logistics and internal operations of a program may affect the quality of state funded prekindergarten programs. Variation in quality of programs likely affects the long-term impacts of prekindergarten. Future research could explore the quality of state funded prekindergarten programs and study how variation in program quality and structure affects the long-term impacts for different student populations.

## Data Availability

The data sets generated and/or analyzed during the current study are part of ongoing program evaluation activates by the state of New Mexico and are not publically available due to data protections put into place by the federal government and the state of New Mexico. Contact the corresponding author for more information. Note that this research was performed as a part of a government evaluative study using de-identified administrative data and the study was not experimental. Further descriptive information is available in the original legislative report found here: https://www.nmlegis.gov/Entity/LFC/Documents/Program_Evaluation_Reports/Prekindergarten%20Quality%20and%20Educational%20Outcomes.pdf.

## Abbreviations

NAEP: National Assessment of Educational Progress
FRL: Free or Reduced-Price Lunch
PPP: Perry Preschool Project
CCPCP: Chicago Child–Parent Center Program
NIERR: National Institute for Early Education Research
NJAPP: New Jersey’s Abbott Preschool Program
BPK: Boston Prekindergarten
EEEP: Maryland’s Extended Elementary Education Program
NCMF: North Carolina More at Four
NMPK: New Mexico’s prekindergarten
MCAS: Massachusetts Comprehensive Assessment System
TOUP: Tulsa Oklahoma’s universal prekindergarten program
PED: Public Education Department
CYFD: Children, Youth and Families Department
ECECD: Early Childhood Education and Care Department
NIEER: National Institute for Early Education Research
NMLFC: New Mexico Legislative Finance Committee
SBA: New Mexico Standards-Based Assessment
PARCC: Partnership for the Assessment of Readiness for College and Career
PSM: Propensity score matching

## Footnotes

1 Moderate improvements in working memory and impulse control may also contribute to students’ academic gains.

2 During the 2005 regular legislative session, the New Mexico legislature established the state prekindergarten program through the Pre-Kindergarten Act (New Mexico Laws 2005, Chapter 170; House Bill 337).

3 New Mexico’s prekindergarten program only served four-year-old children from FY06 through FY15. In 2015, New Mexico enacted pilot initiatives to expand prekindergarten services to three-year-old children through the CYFD and offer extended day services through the PED. These pilot initiatives have continued to provide expanded prekinderarten services to three-year-old and four-year-old children each year since 2015.

4 During the 2019 regular legislative session, the New Mexico Legislature established the through the Early Childhood Care and Education Department Act (New Mexico Laws 2019, Chapter 48; Senate Bill 22).

5 The New Mexico state fiscal year runs from July 1 to June 30 corresponding with the same school year.

6 After transitioning from the SBA to the PARCC, New Mexico has since moved away from the PARCC to a new standards based assessment although due to the pandemic, complete statewide testing on the assessment has not occurred since school year 2018–2019.

7 The state's Pre-Kindergarten Act, NMSA 1978 § 32A-23–6 (2005) called for prekindergarten proposals to be "…evaluated on the percentage and number of public elementary schools in the community that are not meeting the proficiency component required for calculating adequate yearly progress and that are serving children, at least sixty-six percent of whom live within the attendance zone of a Title 1 elementary school.”.
